# Editorial for the Special Issue “Selected Papers from the ISTEGIM’19—Thermal Effects in Gas Flow in Microscale”

**DOI:** 10.3390/mi11090879

**Published:** 2020-09-21

**Authors:** Lucien Baldas, Jürgen J. Brandner, Gian Luca Morini

**Affiliations:** 1 Institut Clément Ader (ICA), Université de Toulouse, CNRS-INSA-ISAE-Mines Albi-UPS, 31400 Toulouse, France; 2 Institute of Microstructure Technology, Campus Nord, Hermann-von-Helmholtz-Platz 1, 76344 Eggenstein-Leopoldshafen, Germany; 3 Microfluidics Laboratory, Department of Industrial Engineering (DIN), University of Bologna, Via del Lazzaretto 15/5, 40131 Bologna, Italy

MIGRATE (www.migrate2015.eu) was an H2020 Marie Skłodowska-Curie European Training Network running from November 2015 to October 2019, intended to address some of the current challenges to innovation that face European industry with regard to heat and mass transfer in gas-based micro-scale processes. This project had 10 participants and 6 associated partners coming from all over the European community. It covered different aspects of enhanced heat transfer and thermal effects in gases: from modeling of heat transfer processes and devices, development and characterization of sensors and measurement systems for heat transfer in gas flows, and thermally-driven micro gas separators, to micro-scale devices for enhanced and efficient heat recovery in environmental applications, transport, telecommunications and energy generation. In the framework of this MIGRATE project, a 2-day symposium, ISTEGIM’19–Thermal Effects in Gas flow in Microscale, was held on 24–25 October 2019, in Ettlingen, Germany, with the aim to showcase the main achievements of the project and to exchange information on the covered topics with the wider scientific community. This Special Issue collects 11 papers (10 research articles and one review) presented during the symposium by members of the MIGRATE Network, by invited speakers and by contributing authors.

Two of these papers deal with fundamental issues encountered in microfluidic systems involving gases. Understanding gas–solid surface interactions under rarefied conditions is of primary importance for efficient modeling of gas flows and heat transfer in microsystems. Mohammad Nejad et al. [1] used a molecular dynamics approach to simulate the behavior of two monoatomic gases, argon and helium, confined between two gold surfaces, kept at different temperatures in order to determine the corresponding energy and momentum accommodation coefficients. They showed that the pressure level has a minor influence on the accommodation coefficients value. On the contrary, by comparing the simulation results with empirical and numerical values in the literature, the gas–solid interatomic potential based on ab-initio calculations was shown to be more reliable for computing accommodation coefficients than the Lorentz–Berthelot and Fender–Halsey mixing rules. In liquid desiccant dehumidification systems, the thermal effect induced by interfacial phase change affects the spatio-temporal evolution of the mass flux at the liquid–air interface. Wang et al. [2] investigated experimentally, using optical imaging and infrared (IR) thermography, the thermal effects during vapor absorption into hygroscopic liquid desiccant droplets. They showed that the substrate conductivity strongly affects the transient heat transfer process. In addition, the temperature change during vapor absorption was shown to be much lower than that induced by the concentration change, which permits the possible application of a simplified isothermal model to capture the main mechanisms during vapor absorption into hygroscopic droplets.

Local experimental data are of crucial importance for the characterization of liquid and gaseous flows in microchannels. As highlighted in the review by Nachtmann et al. [3], molecule-sensitive, spatially-resolved technologies (e.g., Raman spectroscopy or fluorescence techniques) which combine spatial and temporal resolution with molecular selectivity and no disturbance of fluidics, can be efficiently applied for monitoring and measuring in microchannels. Advantages and disadvantages of the current state-of-the-art approaches are analyzed in that review and guidelines are proposed for the selection of a suitable measuring system according the application. In Fratantonio et al. [4] molecular tagging velocimetry (MTV) by direct phosphorescence, another non-invasive optical measurement technique, is applied to argon and helium rarefied flows in a millimetric channel, providing the first flow visualizations ever reported of a confined gas flow in the slip regime (for Knudsen numbers down to 0.014). Despite the new challenges encountered in carrying out local velocity measurements in gas flows characterized by high molecular diffusion, this work demonstrated that MTV is currently the most promising technique for providing direct measurements of the slip velocity at the wall in rarefied gas flows. Gas–surface interaction plays an important role in the heat transfer from a hot to a cold surface in rarefied conditions. Yamaguchi and Kito [5] measured, in the free-molecular to near free-molecular flow regimes, heat fluxes from anodic oxide films on aluminum with different anodizing times through a gas confined between two surfaces with different temperatures, to extract the energy accommodation coefficient (EAC). They showed that the EAC increased with the surface roughness, which depends on the duration of the anodization process and which was observed by a scanning electron microscope. Velocity measurements of two-phase flows in microchannels, which play an important role in the design of portable devices for biological and chemical samples analysis are also an open issue due to the lack of low-cost detection systems. An approach based on a low-cost optical signal monitoring setup was used by Gagliano et al. [6] to investigate the slug flow generated by the interaction of two immiscible fluids (air and water) in two serpentine microchannels with diameters of 320 and 640 µm. The optical signal obtained from a photo-diode set-up was analyzed in real-time to obtain water and air slug velocity and frequency. The successful implementation of this real-time detection platform in LabVIEW was demonstrated, paving the way to the possible development of non-invasive, low-cost portable systems for micro-flow analysis which could also be suitable for an easy on-chip integration.

Heat transfer management is one of the major applications of fluidic microsystems. The numerical design of micro heat exchangers is a process consuming large amounts of computational resources due to the complexity of the device geometry (the inlet and outlet manifolds and the large number of parallel microchannels). Rehman et al. [7] proposed a novel hybrid numerical methodology in which the microchannels are modeled as a porous medium where a compressible gas is used as the working fluid. This reduced order model, based on a modified formulation of the Darcy–Forchheimer law to consider the compressible nature of gas flow in microchannels, was used to develop a complete model of a double layer micro heat exchanger with collecting and distributing manifolds and was validated by comparison with experimental data. The adopted hybrid methodology resulted in a savings of at least 20 million computational nodes compared to the meshing strategy adopted in a classic conjugate heat transfer numerical analysis. The high performances of micro heat exchangers in terms of heat transfer rates are due to the high surface to volume ratios linked to the small channel dimensions, but can also be improved by the generation of local turbulences in the microchannels thanks to perturbators. Joseph et al. [8] used a reduced order numerical model based on a porous medium approximation for the microchannels, to analyze flow maldistribution and pressure losses for two different perturbators: wire-net and S-shape perturbators. The wire-net microchannels showed a better performance in terms of thermal efficiency, while the S-shape fins provided better performance in terms of pressure loss. Experimental tests were performed on a single microchannel with S-shape perturbators and a micro heat exchanger with triangular collectors for the validation of the numerical simulations; the results showed that the perturbators provoke an earlier laminar to turbulent flow transition. In the last few decades, miniaturized regenerative cryocoolers found applications in various domains such as IR imaging systems, aerospace applications and cryosurgery, to cite a few. In these closed-cycle cryocoolers, the regenerator is a key component for the overall performance of the system. Almtireen et al. [9] developed a one-dimensional numerical model, based on the discretization of the ideal regenerator thermal equations, to study the thermal interaction between the working gas and the metallic porous medium for various geometric and operating conditions. A 2D axisymmetric Computational Fluid Dynamics (CFD) model was also built on ANSYS Fluent to evaluate the validity of the ideal regenerator model. Even if the 1D ideal model was capable of providing rather good predictions of the thermal interactions between the gas and the matrix material, it was shown that more accurate results provided by a 2D CFD model or a non-ideal 1D model are required for an efficient design or for the optimization of the regenerator element.

The last series of papers published in this Special Issue are devoted to more complex microsystems integrating multiple components on the same chip, for different types of applications. High-order harmonic generation (HHG) beams produced by ultrashort laser pulses focused in a gaseous medium are commonly exploited in the fields of extreme ultraviolet spectroscopy and attosecond science. However, due to their technological complexity, these HHG-based coherent light sources stayed confined, up to now, within a few advanced laboratories. In this context, Ciriolo et al. [10] presented the application of femtosecond laser micromachined complex glass microchips to the generation of high-order harmonics in gas. In their device, a microchannel filled with gas through a 3D network of gas distribution microchannels acts as a hollow waveguide for the driving laser. A considerable increase in harmonics generation efficiency was observed, compared with standard harmonic generation in gas jets; this opens new perspectives for the downscaling of an entire HHG-based beamline to a glass chip with numerous additional functionalities. There is currently a high demand for compact, portable, accurate and rapid gas detectors for indoor air monitoring. In this framework, Mariuta et al. [11] worked on the development of a miniaturized detector of formaldehyde (HCHO), which is one of the major indoor airborne pollutants. The proposed sensor is based on the Hantzsch reaction which occurs after HCHO is trapped in the liquid phase, coupled to the fluorescence optical sensing methodology. On-chip integration of the detection part of the sensor was presented in this paper. A fluorescence detector, based on an LED-induced fluorescence technique coupled to a CMOS image sensor as an ultra-low light detection system, was developed and proved to be capable of detecting 10 µg/L of formaldehyde derivatized into 3,5–diacetyl-1,4-dihydrolutidine (DDL) in a 3.5 µL interrogation volume.

A large part of the results reported in this Special Issue was obtained in the framework of the PhD theses of the students enrolled in the MIGRATE European Training Network. Other works presented in this issue also come from research groups in touch with the partners of the MIGRATE network. The brilliant results obtained by the MIGRATE early stage researchers during their work within a structured European training network demonstrate that, with efficient international cooperation among research centers and companies active in microfluidics, it becomes possible to enhance the research quality and accelerate the transition between fundamental research and applications. In addition, the network acts as catalyst of fruitful links with new partners on common topics. We think that the papers collected in this Special Issue demonstrate the strength of the Marie Skłodowska-Curie Actions promoted by the European Commission.

We wish to thank all authors who participated to the ISTEGIM’19 symposium and submitted their papers to this Special Issue. We would also like to acknowledge all the reviewers for dedicating their time to provide careful and timely reviews to ensure the quality of this Special Issue.
